# Comprehensive Evaluation of Peripheral Nerve Regeneration in the Acute Healing Phase Using Tissue Clearing and Optical Microscopy in a Rodent Model

**DOI:** 10.1371/journal.pone.0094054

**Published:** 2014-04-08

**Authors:** Yookyung Jung, Joanna H. Ng, Cameron P. Keating, Prabhu Senthil-Kumar, Jie Zhao, Mark A. Randolph, Jonathan M. Winograd, Conor L. Evans

**Affiliations:** 1 Wellman Center for Photomedicine, Harvard Medical School, Massachusetts General Hospital, Boston, Massachusetts, United States of America; 2 Plastic Surgery Research Laboratory, Harvard Medical School, Division of Plastic and Reconstructive Surgery, Department of Surgery, Massachusetts General Hospital, Boston, Massachusetts, United States of America; University of Edinburgh, United Kingdom

## Abstract

Peripheral nerve injury (PNI), a common injury in both the civilian and military arenas, is usually associated with high healthcare costs and with patients enduring slow recovery times, diminished quality of life, and potential long-term disability. Patients with PNI typically undergo complex interventions but the factors that govern optimal response are not fully characterized. A fundamental understanding of the cellular and tissue-level events in the immediate postoperative period is essential for improving treatment and optimizing repair. Here, we demonstrate a comprehensive imaging approach to evaluate peripheral nerve axonal regeneration in a rodent PNI model using a tissue clearing method to improve depth penetration while preserving neural architecture. Sciatic nerve transaction and end-to-end repair were performed in both wild type and thy-1 GFP rats. The nerves were harvested at time points after repair before undergoing whole mount immunofluorescence staining and tissue clearing. By increasing the optic depth penetration, tissue clearing allowed the visualization and evaluation of Wallerian degeneration and nerve regrowth throughout entire sciatic nerves with subcellular resolution. The tissue clearing protocol did not affect immunofluorescence labeling and no observable decrease in the fluorescence signal was observed. Large-area, high-resolution tissue volumes could be quantified to provide structural and connectivity information not available from current gold-standard approaches for evaluating axonal regeneration following PNI. The results are suggestive of observed behavioral recovery *in vivo* after neurorrhaphy, providing a method of evaluating axonal regeneration following repair that can serve as an adjunct to current standard outcomes measurements. This study demonstrates that tissue clearing following whole mount immunofluorescence staining enables the complete visualization and quantitative evaluation of axons throughout nerves in a PNI model. The methods developed in this study could advance PNI research allowing both researchers and clinicians to further understand the individual events of axonal degeneration and regeneration on a multifaceted level.

## Introduction

Improving treatment outcomes after peripheral nerve injury (PNI) remains an important goal for researchers and clinicians worldwide. With over 300,000 annual upper extremity injuries, approximately 100,000 surgical repairs in Europe alone [1], [2], and an increasing incidence of blast injuries sustained during warfare [3], [4], there is a recognized and compelling need to accelerate peripheral nerve regeneration techniques and technologies; this has resulted in the development of peripheral nerve microsurgery in the 1960s and the subsequent refinement of a range of surgical techniques and treatments [5], [6]. Despite these advances, motor outcomes for patients remain delayed, unpredictable and usually incomplete [1], [2], [3].

Optical imaging has historically played an important role in guiding the development of new nerve repair methods. Unlike behavioral and physiological methodologies of measuring nerve recovery, which include histomorphometry, electrophysiology, and walking track analysis, imaging nerves and axons provides comprehensive and direct information about the intricacies of the neural environment [7], [8]–[10]. Direct visualization of the regenerative and degenerative fronts after neurorrhaphy remains advantageous compared with current outcomes measurements of nerve regeneration. For example, histology is a current method that provides quantitative data regarding axon quantity and diameters - parameters that improve with regeneration - but is labor-intense and requires destructive sectioning of the three-dimensional architecture of the nerve [10]. Information acquired from histology is cross-sectional in nature and, unfortunately, longitudinal information regarding axonal branching from the repair site and muscle re-innervation is lost. Additionally, results obtained from histology do not always correlate well with behavioral recovery of function, such as walking track analysis to calculate sciatic function index [11]. Measurements of compound nerve action potentials (CNAP) in small animals can be difficult due to the inherent short distances between probe sites [11]; often, there exist several different neural pathways to carry the same evoked electrical current to a distal target other than the pathway of interest.

In recent years, the use of advanced optical microscopy methods for imaging genetically engineered transgenic animal models has markedly improved our ability to study hindlimb [12] and craniofacial nerves [13]. Confocal, multiphoton, coherent anti-Stokes Raman scattering (CARS) microscopies, and optical coherence tomography (OCT), have allowed researchers to directly visualize the regenerative and degenerative environment after PNI with subcellular resolution, and can be performed *in vitro*, *ex vivo*, and *in vivo* [7]. Both multiphoton and confocal microscopy have been utilized extensively to image fluorophore-labeled nerves and axons, either via exogenous agents or through genetically incorporated fluorescent proteins. Re-innervation of superficial peripheral nerves in the dermis as well as neural sprouting in the central nervous system (CNS) has been captured using these modalities [14], [15]. For example, when applied *in vivo*, the tendency of axons to compete for the same end target and the tremendous plasticity of nerves has been illustrated in the neonatal period at the neuromuscular junction [8], [9]. CARS microscopy, a chemically selective imaging technology, has allowed for visualization of myelin sheaths in fresh nerve tissue *in vivo*, without the need for antibody labeling or chromophore markers [16]–[19]. This has given researchers the ability to track both myelin degradation and regeneration after sciatic nerve crush injury [19], [20] as well as white matter degeneration in the central nervous system [21].

Despite their utility, optical imaging techniques can suffer from limited imaging depth penetration due to scattering of light within the turbid environment of most biological tissues [18], [22]. As light passes through tissue such as nerves, photons are scattered due to particles, layers, and other inhomogeneities that alter the local index of refraction, resulting in a perturbed focal spot geometry, reduced power at the focal plane, and signal loss due to emission signal attenuation. Nerve bundles, both central and peripheral, have particularly large scattering coefficients [22], limiting visualization of nerves to the most superficial layers ranging from a few tens of microns for confocal and CARS microscopy up to 300 micrometers deep for two-photon microscopy and OCT [17], [22], [23]. For comparison, a rat sciatic nerve is consistently >1000 micrometers in diameter. The inability to visualize nerves beyond these depths leads to insufficient knowledge of the structural and mechanistic events that occur throughout nerves during axonal regeneration. Without deep imaging capabilities, key information remains unavailable, such as the manner of individual axonal regrowth through the repair site and the percentage of axons ultimately innervating their distal target sites. Access to this deep structural data at the site of nerve injury could aid in identifying novel surgical nerve repair techniques.

A process known as tissue or optical clearing offers a method to overcome this limitation. Tissue clearing is a procedure that can reduce tissue light scattering and dramatically improve depth penetration through the infusion of chemicals into tissue that act to homogenize the index of refraction. While some tissue clearing methods have utilized glycerin to perform this “index matching” process, the most effective means to clear tissue involves a combination of dehydration along with the solvation of lipids within the sample using a sequence of solvents [24]. This method, which has been used to improve depth penetration in solid organs with confocal microscopy [25], was recently utilized to successfully visualize whole axons in large spinal cord segments in transgenic thy-1 green fluorescent protein (GFP) mice using standard two-photon microscopy [25], [26]. Imaging depth penetration was improved to more than 1300 micrometers compared to non tissue-cleared images [26], [27]. Importantly, this approach preserved neural structural integrity and did not significantly reduce the emission signal from GFP in the axons.

With the advent of new treatments, microsurgical techniques such as end-to-side repair [6], [28], [29], and the application of devices such as nerve conduits and wraps, obtaining a clear picture of peripheral nerve repair on the microscale is more important than ever. Comprehensive microscopic evaluation of nerve regeneration, supported by different imaging modalities, has the capacity to unravel previously unknown structural and biological details. Improving imaging penetration depth via tissue clearing has the ability to guide research and provide the means to monitor treatment outcomes, enable fundamental studies into optimal methods of nerve repair, and accelerate the rate at which peripheral nerve research is performed. In this study, we demonstrate comprehensive evaluation of neural degeneration and regeneration, using tissue clearing and confocal imaging, in a well-established rat animal model for PNI. We demonstrate both qualitative evaluation and quantitative analysis of whole sciatic nerves in three dimensions, including myelin degradation and the neural environment after transection injury, illustrating individual axonal events after neurorrhaphy that would have otherwise been missed.

## Materials and Methods

### Ethics Statement

Experiments were carried out in accordance with recommendations from the National Institutes of Health *Guide for the Care and Use of Laboratory Animals*. The animal protocols and experiments were approved by the Massachusetts General Hospital Institutional Animal Care and Use Committee (IACUC) (Animal welfare assurance # A3596-01). All efforts were made to minimize the number of animals used. Surgeries were carried out under sodium pentobarbital anesthesia, and all efforts were taken to minimize animal suffering.

### Subjects

A total of four transgenic thy-1 GFP male rats (University of Toronto) and five non-GFP wild-type Sprague Dawley male rats (Charles River Laboratories), all weighing 300–350 g, were used for the imaging portion of this study. All animals were housed in single cages with unlimited access to food and water *ad libitum*. All animals underwent right sciatic nerve transection and primary end-to-end repair with standard microsurgery in the designated laboratory rodent procedure room. The sciatic nerves from the non-GFP group were harvested at baseline, 3, 6, 9 and 12 days after repair (n = 5), and underwent antibody staining to label individual axons. All nerves were then imaged with confocal microscopy to confirm successful tissue staining. They were then imaged with CARS microscopy at three points (5 mm proximal to, at, and 5 mm distal to the repair site) to evaluate axonal myelin sheaths. All nerves then underwent tissue clearing *ex vivo*, followed by imaging with confocal microscopy at the same 3 points on the nerve to assess the degree of depth penetration, the preservation of axonal architecture, the quality and preservations of the antibody stain, and the degree of axonal degeneration and regeneration.

Sciatic nerves from thy-1 GFP rats were harvested at baseline, postoperative days 2, 4, and 22 (n = 4), and processed in a manner similar to the wild-type nerves to serve as a comparison group without the need for immunofluorescence staining. Each image of the sciatic nerve from thy-1 GFP rats acquired with confocal microscopy was visually assessed, and subsequently subjected to quantitative analysis.

For the non-imaging arm, a separate group of eight wild type Sprague-Dawley male rats, all weighing 300–350 g, was utilized for functional recovery studies following transection and microsurgical neurorrhaphy.

### Surgery

All rats were acclimated to their environment for a minimum of 2 days. Each rat received one preoperative dose of intramuscular buprenorphine (0.01–0.03 mg/kg) 30 minutes prior to administration of general anesthesia with intraperitoneal sodium pentobarbital (50 mg/kg), recommended by the MGH IUCAC. Once adequately asleep, the right hindleg was shaved and prepped with 10% povidone iodine prep solution (Medline). A dorsolateral gluteal muscle splitting incision was performed, and the sciatic nerve was exposed from the sciatic notch to the distal trifurcation. This was isolated from surrounding connective tissue and the epineurium adequately exposed. The nerve was sharply transected with a straight microscissor (F.S.T, Germany) approximately 1 cm distal to the sciatic notch and the epineurium primarily approximated with 5–6 circumferentially placed 10–0 nylon sutures (Ethicon) in an end-to-end fashion. The muscle and skin were then closed in a multilayer fashion with 4–0 vicryl and 4–0 monocryl suture (Ethicon), respectively. Triple antibiotic ointment (Curad) was applied generously to the incision and the rat was observed until awake. Bitter apple (Grannick's) was applied to deter automutilation. Postoperative analgesia was administered every 12 hours for a total of 72 hours with intramuscular buprenorphine (0.01–0.03 mg/kg). At specified time points, rats underwent euthanasia with intraperitoneal sodium pentobarbital (200 mg/kg). The right hindleg was then shaved and the same incision was entered. The sciatic nerve was exposed and carefully dissected from surrounding tissue and adhesions (scar tissue). An approximately 1.5 cm long segment was harvested that included the suture site.

### Confocal Imaging System

An Olympus Fluoview FV1000 automated laser scanning confocal was used for all imaging experiments. The microscope utilized an IX81 base. A 20×0.75 NA objective lens was used for the large area whole nerve imaging, and a 40×0.8 NA long working distance (LUMPFl) objective was used to visualize detail in the tissue and provide quantitative analysis. A programmable, automated translation stage covering over 10 cm of length with submicrometer accuracy was used for high resolution, large area mosaic imaging at designated time points [30]. GFP was excited at 488 nm and its emission collected between 500 and 600 nm. AlexaFluor-647 labeled secondary antibodies were excited at 635 nm with emission collected between 650 and 750 nm.

### CARS Microscopy

A portion of the output of a 1064 nm, 7-ps laser (picoTRAIN, HighQ) was converted to 817 nm via a sync-pumped tunable optical parametric oscillator (Levante, APE) as described previously [17]. The 817 nm pulse train served as the pump, and was combined with the original 1064 nm “Stokes” pulse train using a dichroic mirror along with a delay stage to ensure that the pulse trains were temporally overlapped. The energy difference between 817 and 1064 nm beams was set to correspond to the CH_2_ symmetric stretching vibration energy of the lipids found in myelin. The combined train of laser pulses was introduced into the side-port of the confocal FV1000 scanhead (described above) for routine imaging. The epi-CARS signal generated from tissue was collected at the IX81 sideport, filtered through two 650 nm, 25 nm bandpass filters, and detected using an H7422-40 photomultiplier tube (PMT, Hamamatsu). The signal output of the PMT was amplified using a high-speed current amplifier (Femto, HCA-4M-500K) and introduced into an Olympus input/output box for data collection.

### Two Photon Imaging

An Olympus Fluoview FV1000MPE multiphoton microscope was used for all two photon imaging experiments. This microscopy was outfitted with an IX81 inverted stage. A titanium:sapphire laser (SpectraPhyiscs, DeepSee) provided tunable femtosecond pulses, and was configured to be automatically controlled by the Olympus software. GFP was excited at 820 nm, with the emission collected between 495 and 540 nm on built-in non-descanned detectors.

### Immunofluorescence Staining

The whole mount immunofluorescence staining protocol was developed with the Wellman Center photopathology laboratory. Excised nerves were first fixed overnight with 4% paraformaldehyde solution at 4°C. The next day nerves were treated with a 50% methanol solution in water for 20 minutes, followed by a 20 minute incubation in phosphate buffered saline (PBS). Nerves were then permeabilized with 1% Triton X-100 in PBS three times for 20 minutes each, blocked with blocking buffer (1% Triton in PBS, 10% Fetal Bovine Serum, 0.2% Sodium Azide) for 1 hour twice, washed with blocking buffer twice for 5 minutes each, and then incubated with the primary, 160 kDa anti-neurofilament mouse monoclonal antibody (Invitrogen, #13-0700) for 3 days on a rocker at 4°C. After 3 days, the samples were placed at room temperature (25°C) for 30 minutes, washed with 1% Triton in PBS three times for 3 minutes each, blocked with blocking buffer three times for 1 hour each, washed with 1% Triton in PBS three times for 3 minutes each, and then incubated with secondary goat anti-mouse IgG antibody conjugated to AlexaFluor-647 (Invitrogen, #A-21235) for 3 days. Finally the specimens were washed with PBS three times for 5 minutes each, 1% Triton in PBS three times for 15 minutes each, and PBS three times for 5 minutes each to remove excess AlexaFluor-647.

### Tissue clearing

The tissue clearing protocol was adapted from Ertürk *et al*. 2011 [25]. The nerve was immersed in a stepwise fashion to dehydrate any water molecules, first in 50% tetrahydrofuran (THF):distilled water (dH_2_O) solution for 30 minutes, followed by 80% THF:dH2O for 30 minutes, then 100% THF three times for 30 minutes each. It was then immersed in 100% dichloromethane (DCM) for 20 minutes to remove lipid content. The final step was immersion in a mixture of benzyl alcohol and benzyl benzoate at a ratio of 1∶2 for 5–10 minutes in order to make the nerve transparent.

### Quantitative data analysis

A set of confocal microscopy images were analyzed to follow nerve degeneration and regeneration. Matlab and ImageJ software equipped with the LOCI toolkit [31] were used for imaging analysis. Volumetric images of axons were acquired so that the xy plane was parallel to the longitudinal direction of axonal propagation. As axons were relatively sparse within the volume of the nerve, image volumes were z-stacked prior to calculations for more efficient analysis. Both whole and fragmented axons were measured to occupy a space approximately 25 micrometers thick over distances of 200 um; this height was used for all z-stacks. To build these projected images, 13 2D-slices were summed at 2 μm intervals along the z-axis. A single data set for image analysis was derived from a tissue dimension of 200×200×24 μm^3^. 32 data sets with the same dimensions were taken from random areas 1∼2 mm distal to the repair site at various depths. The stacked images were first transformed into binary images by setting a threshold to remove background noise, followed by the creation of boundaries to outline each axonal segment. Eccentricity, length, and area of the axonal segments were used to create a threshold to distinguish between long (healthy) and short (degenerated) fragments. To be sorted as long fragments, the recognized segments in the images had to satisfy all of the following conditions: eccentricity >0.99, major axis >200 pixels, and total filled area >2000 pixels. Short fragments were those that did not meet the above criteria. Once all segments were sorted into long or short according to the above criteria, the total number of pixels that comprise the sorted segments was counted, representing the quantity of both long and short fragments. This value was normalized to the total number of pixels of all unsorted segments.

### Walking track analysis

To assess functional recovery, animal hindlimbs were dipped in dilute India Ink and the animal was allowed to scamper up a two foot long wooden ramp lined with white paper in designated laboratory space. Three clear consecutive inkprints on the injured leg were measured for planar length, toe spread (distance between first and fifth toes), and intermediary toe spread (distance between second and fourth toes), and compared to the non-injured leg to calculate sciatic functional index (SFI), as previously described by Bain et al. [32]. SFI was recorded at baseline, 1 week and bi-weekly thereafter for 12 weeks after neurorrhaphy.

## Results

### Tissue clearing enables imaging of individual axons throughout the sciatic nerve

As previously mentioned, light scattering caused by the numerous heterogeneities in nerve optical refractive index leads to poor depth penetration for many optical microscopy tools. Even when two-photon microscopy is used, the maximum reported depth penetration in nerves in transgenic animal models is 290 μm [26]. Following tissue clearing, however, the depth penetration can be increased beyond 1 mm (Video S1a). As the thickness of rat sciatic nerves is approximately 1 mm in diameter, confocal imaging following tissue clearing allowed for complete fluorescence imaging throughout entire nerve bundles. Importantly, the tissue clearing protocol did not affect the immunofluorescent labeling, with axons retaining strong fluorescence signals following the procedure.

**Figure 1:**
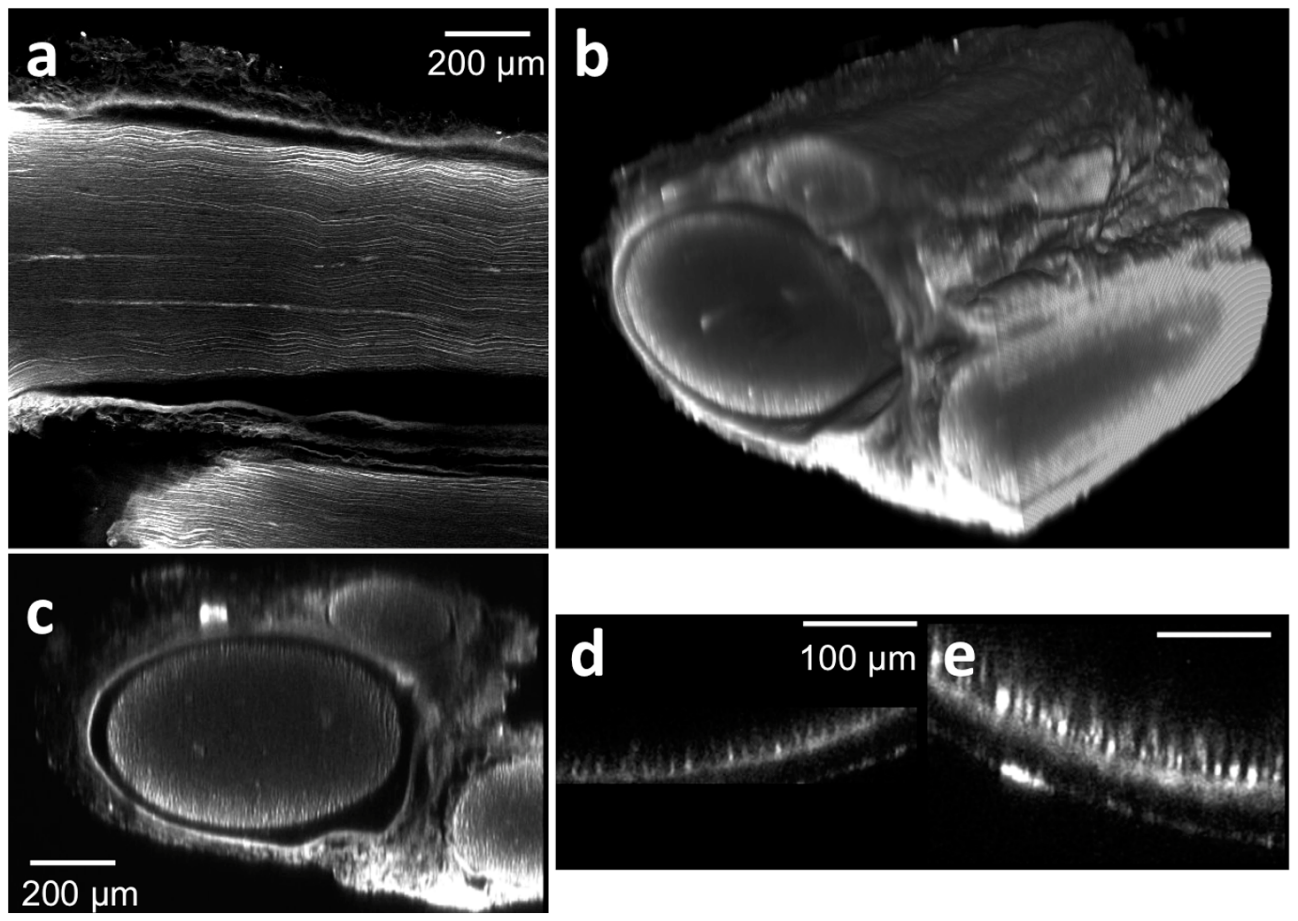
Tissue clearing following antibody staining allows for visualization of axons throughout entire rat sciatic nerves. Tissue were stained with anti-neurofilament antibodies using a whole mount immunofluorescence method. (a) A lateral (xy) confocal image at the depth of approximately 400 μm from the surface of a stained and cleared wild type rat sciatic nerve. (b) Three dimensional view of a whole stained and cleared sciatic nerve. Non-specific GFP epineurial staining can also be seen secondary to presence of fibroblasts, collagen, and vasa nervorum in the epineurium, which does not affect analysis. (c) Reconstructed transverse cross sectional image. Three fascicles surrounded by the epineural sheath can be recognized. For comparison, a transverse cross sectional image of non-tissue cleared, stained sciatic nerve is shown in (d); note that non-specific staining occurs in the epineurial sheath as well, but only the right portion of the sheath is observed. (e) Two-photon transverse cross sectional image of a sciatic nerve from a transgenic rat (thy-1 GFP).

Figure 1a shows a lateral (xy) confocal fluorescence image, taken approximately 400 μm deep in the tissue, of a healthy sciatic nerve from a wild-type rat after whole mount immunofluorescence staining followed by tissue clearing. After acquiring xy images at different depths throughout the whole nerve sample, a three dimensional rendering of the entire nerve was constructed (Fig. 1b, Video S1b). Note that non-neuronal tissue like the epineurial sheath is also fluorescent, a phenomenon that occurs likely due to non-specific background staining of diversified tissue like collagen Type 1 and II, fibroblasts and vasa nervorum, which a Neurofilament-M antibody may bind. To demonstrate the significantly increased imaging depth enabled by tissue clearing, the transverse cross sectional image of a tissue-cleared nerve (Fig. 1c) was compared with a non-tissue-cleared nerve (Fig. 1d). For additional comparison, the sciatic nerve of a transgenic rat (thy-1 GFP) was imaged by two-photon microscopy in Figure 1e. Even when using laser light at 820 nm, the depth penetration reaches at best 100 μm due to the light scattering from the optically heterogeneous nerve tissue.

**Figure 2:**
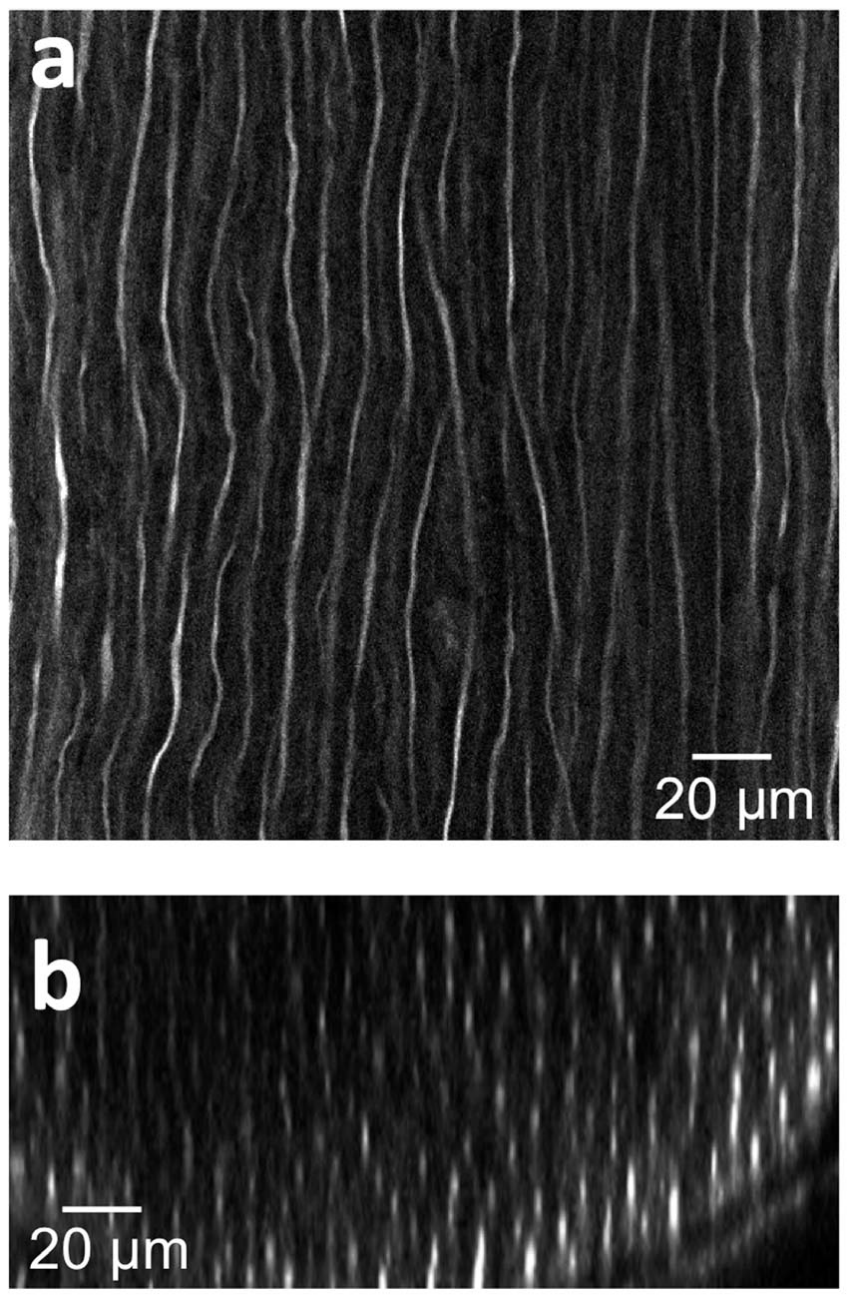
Deep tissue and high resolution images of tissue cleared nerves by confocal microscopy following anti-neurofilament antibody labeling. (a) Lateral (xy) confocal image. (b) Cross sectional image. The elongated shape of the axons in the z direction is caused by the lower relative axial resolution of confocal microscopy.

To demonstrate the high resolution imaging of axons following tissue clearing, 3D image stacks of nerve fibers located within a healthy, wild-type rat sciatic nerve were acquired at 40X magnification (Fig. 2). The image data was processed to construct a three-dimensional volumetric image. Individual axons can be clearly distinguished in both the lateral (Fig 2a) and axial (Fig 2b) planes, demonstrating that unique axons can be both identified and locally traced. The size of each axon as measured by neurofilament staining in tissue cleared nerves is smaller than those found in fresh *ex vivo* samples, and is likely a consequence of the chemical treatments found in both the whole mount staining and tissue clearing processes. As shown in Figure S1, individual axons found in freshly excised tissue are approximately 7∼8 μm in diameter. Following whole mount staining with anti-neurofilament antibodies, the axon size is perceived to be approximately 6 μm (Fig S1e,f). This slight reduction in the axon size is thought to be caused by tissue shrinkage during the fixation process, though it is possible that the neurofilament stain does not represent the entire axonal diameter. Interestingly, the tissue clearing process caused a perceived reduction in the dimension of the neurofilament-stained axons (∼1 μm diameter, Fig S1g,h). However, while the size of the neurofilament bundles decreased, the spacing between neighboring axons did not change significantly, consistent with a previous report in the CNS [25]. Thus, even while the neurofilaments within axons show alteration, tissue clearing is observed to preserve both the overall axonal structure throughout large tissue blocks (Fig. 1c), [25]–[27], and the continuity of the axons themselves (Fig. 2a).

**Figure 3:**
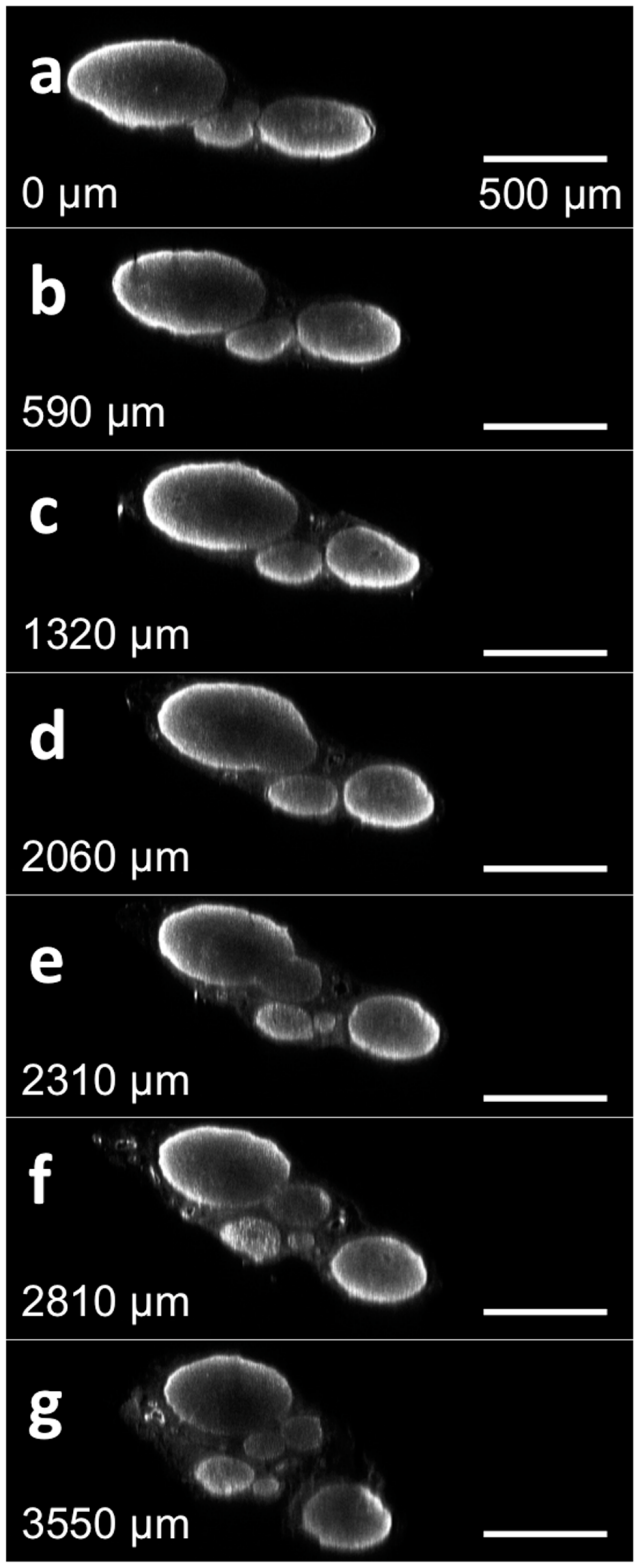
Cross sectional images of a wild-type, stained and tissue cleared sciatic nerve without physical sectioning. The reconstructed cross sectional image of a healthy sciatic nerve from a rat at different locations from a point of reference to the distal end. The distance of each cross section from the initial slice is noted in each subsequent figure. Video S2 contains a flythrough animation.

Figure 3 shows the reconstructed cross sectional image of a wild type, healthy, neurofilament-stained peripheral nerve post tissue clearing. These images show the branching of the proximal nerve first into three distinct, smaller fascicles (Fig. 3). As the nerve continues to branch, these three fascicles further divide into six total fascicles (Fig. 3g). The resolution of these cross sectional images is determined by the axial resolution of the objective lens. In this case, a 20X, .75NA objective was used to acquire image volumes, providing an estimated 2.3 μm of axial (cross-sectional) resolution when 635 nm laser light is used for excitation. As these cross-sectional images can be obtained without physical sectioning, tissue clearing has the potential to enable axonal tracing along the entire nerve from proximal origin near the dorsal root ganglion to distal trifurcation of the nerve near end-organ targets.

### Immunofluorescence staining followed by tissue clearing reveals axonal structure throughout entire peripheral nerves

**Figure 4:**
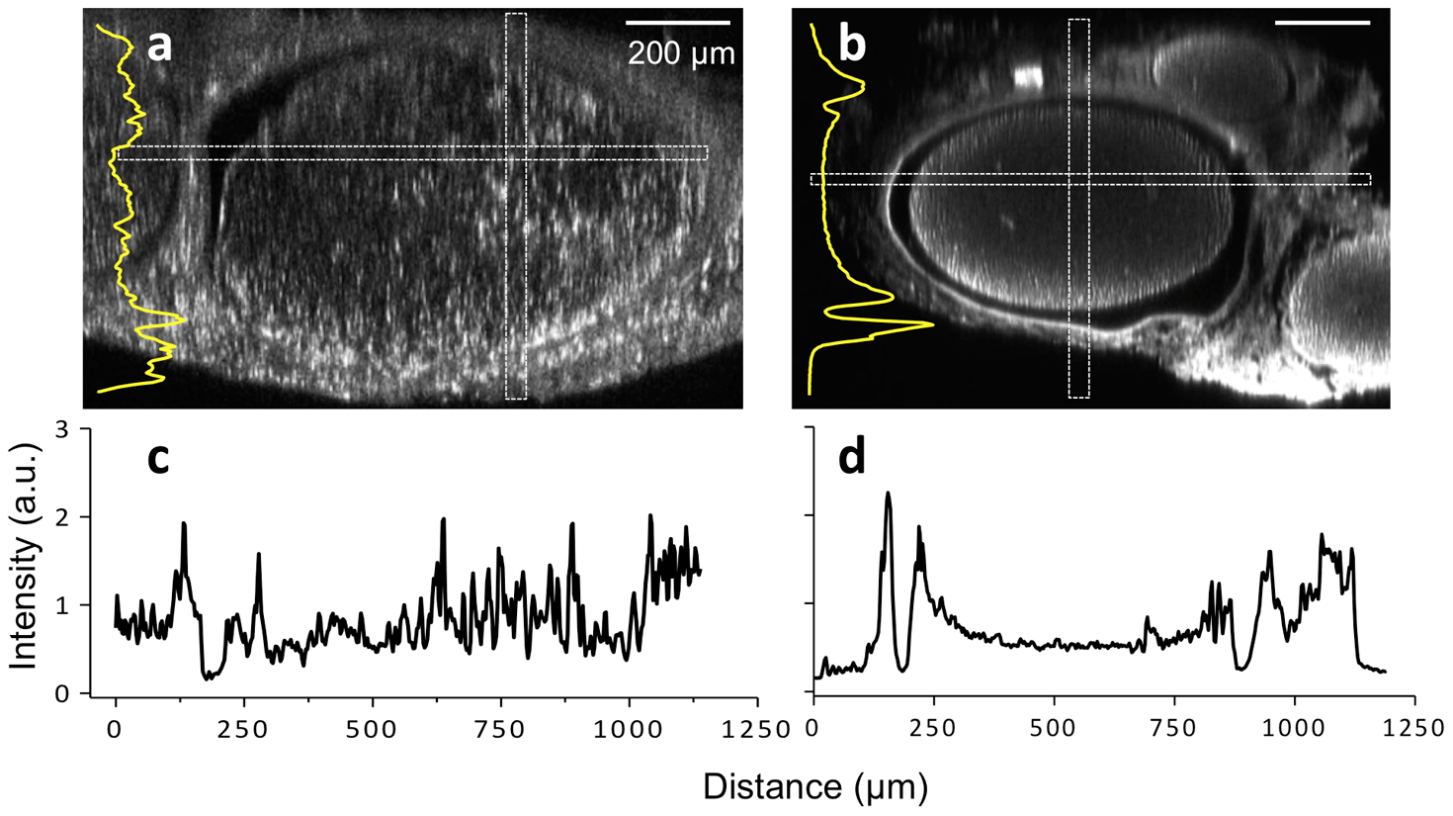
Comparison of the signal intensities between both tissue-cleared GFP and antibody-stained, wild-type sciatic nerves. (a) Tissue-cleared thy-1 GFP nerve (b) Tissue-cleared, wild type nerve following immunofluorescence staining. Note that both (a) and (b) cross sectional images are presented at slightly different magnification. Non-specific GFP labeling can be observed in (a) due to the ubiquitous expression of the thy-1 regulatory gene. Insets in (a) and (b) are intensity profiles of vertically elongated rectangular areas (along the z-axis). (c, d) Intensity profiles of horizontally elongated rectangular areas (along the x-axis) are also shown in images a and b, respectively. The vastly improved penetration depth provided by tissue clearing allows for visualization of axons throughout the nerve.

Tissue-cleared nerves taken from a transgenic thy-1 GFP rat (Fig. 4a) were compared against antibody-stained and tissue-cleared nerves from a wild-type rat (Figs. 4b) in order to validate the ability of antibody staining and tissue clearing to accurately depict axonal structure throughout entire nerves. Note that non-specific GFP staining of non-neuronal tissue occurs in Fig. 4a, a phenomenon that occurs due to the ubiquitous expression of the thy-1 regulatory gene [12], [13]. The insets in Figure 4a and 4b show the z-axis intensity profiles acquired from the rectangular regions defined in both images. For both the GFP and antibody-stained nerves, the fluorescence intensity can be observed to decrease unidirectionally from the bottom to the top of the image stack. This is explained by the fact that while tissue clearing does successfully homogenize the tissue index of refraction, it does not completely eliminate all laser light loss and scattering mechanisms, thus leading to minor, predictable and expected emission intensity loss with increasing imaging depth. It should be noted that antibody-stained nerves were observed to be smaller in diameter after undergoing immunohistochemical staining compared with non-antibody stained nerves.

As expected, the GFP fluorescence intensity from a transgenic rat was relatively uniform throughout the tissue (Fig. 4c). In contrast, excised, stained, and tissue-cleared nerves from a wild-type rat were observed to have greater fluorescence signal intensities in the periphery compared with the deeper central core (Fig. 4d). This decrease in central fluorescence signal in wild type rats is due to the limited penetration of the primary anti-neurofilament antibody during the staining procedure. During development of the staining protocol, it was found that the three day antibody incubation used here led to staining throughout the entire nerve at signal levels that substantially exceeded background noise. Despite this central decrease in overall signal intensity, this signal was still intense and distinct from background noise allowing for high signal-to-noise imaging capable of visualizing individual axons deep within the tissue.

### Axonal degradation and regrowth can be visualized following tissue clearing

**Figure 5:**
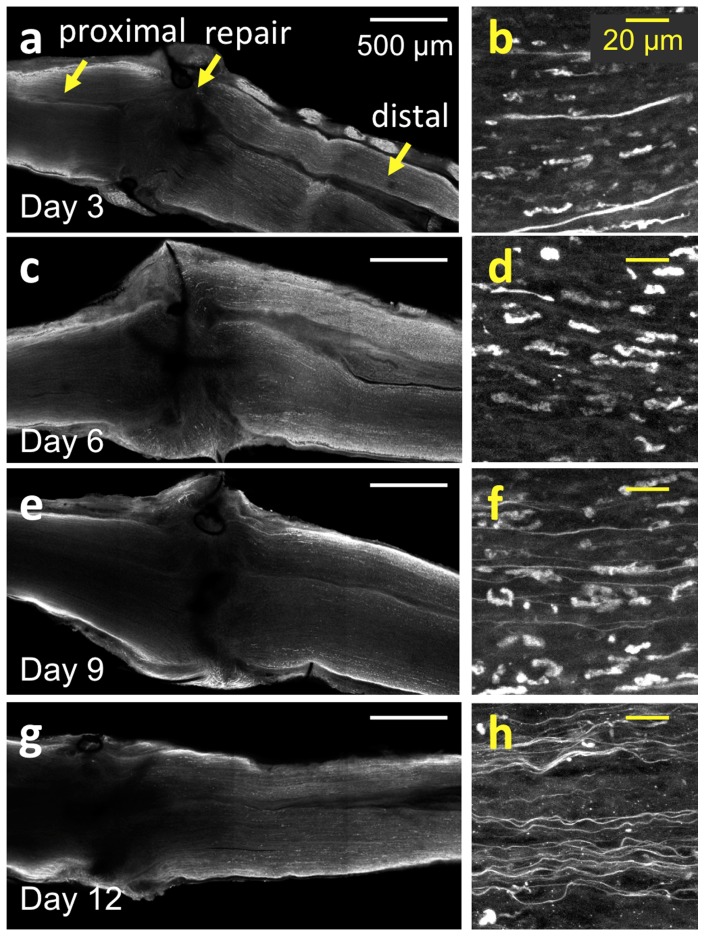
Time course study of sciatic nerves following neurorrhaphy. (a, c, e, g) Large area, *ex vivo* images of wild type rat sciatic nerves that have undergone whole mount immunofluorescence staining and tissue clearing at 3, 6, 9 and 12 days post-neurorrhaphy obtained using confocal microscopy. In the figure, the proximal direction lies to the left of the repair site, while the distal direction lies to the right. (b, d, f, h) High-resolution images of the individual axons located approximately 2 mm to distal direction from the repair site in (a, c, e, g), respectively.

To qualitatively visualize the microscale details of Wallerian degeneration and axonal regeneration throughout whole and intact nerve specimens, the antibody staining and tissue-clearing protocols were applied to wild-type nerves harvested at early timepoints following surgical neurorrhaphy (Fig. 5). Axons throughout the ∼1 mm-thick nerves could be visualized during the different timepoints in the acute healing phase. Confocal images were acquired at depths of 200–500 μm from the epineurial sheath, a depth not accessible using conventional imaging methods (Figs. 5a, c, e, g). As expected from observations in the literature, axons proximal to the site of injury remained connected to their soma and did not undergo degeneration [33]–[35]; these organized, healthy axons can be observed at all time points. Distal to the site of transection, however, axons can be observed undergoing Wallerian degeneration in the days following transection and repair (Video S3a–d). Wallerian degeneration is characterized here by a loss of axonal continuity with presence of ovoid bodies or decrease in fluorescence signal, with axons degrading into debris fragments that follow the original endoneurial tube in the nerve bundle [32], [33]. At three days post-transection, these axonal fragments can be seen concurrently with axons that have yet to degenerate (Fig. 5b), a finding that concurs with the known time course of neuronal events [32], [33]. By six days following surgery, the majority of Wallerian degeneration has ensued, as visualized by a lack of organized axons and an abundance of elliptical or ovoid fragments (Fig. 5d). Degeneration seems to occur by day 3 post-neurorrhaphy and continues to occur through day 9, consistent with known literature in thy-1 GFP rats [12], [13]. At 9 days post-neurorrhaphy, the number of fragments begins to decrease, with a greater number of regenerating axons observed (Fig. 5f). Many regenerated axons can be seen crossing the repair site along with a concomitant decrease of axonal debris at 12 days post transection (Fig 5f). These individual regenerating axons, previously difficult to image due to depth limitation, can be clearly and unambiguously visualized as they cross the suture site. Moreover, the path carved by these axons can be rendered in 3D to map the regeneration process.

### Tissue cleared nerve images can be readily quantified to monitor regeneration

**Figure 6:**
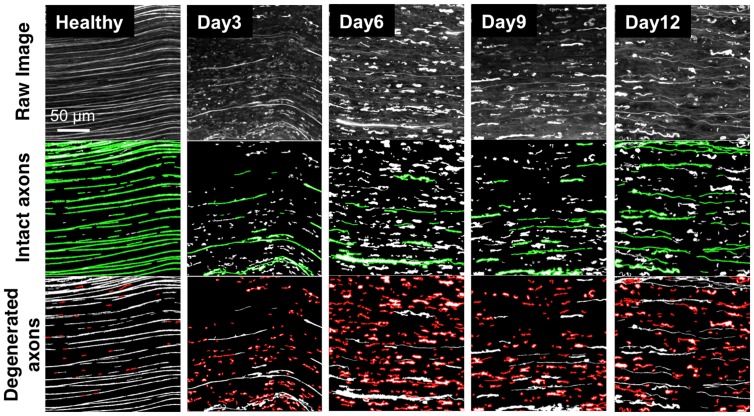
Z-stacked images for quantitative analysis of nerve degeneration and regeneration. Wild type rat sciatic nerves were processed with whole mount immunofluorescence staining and tissue clearing. First row: z-stacked confocal images of tissue cleared sciatic nerves. Second row: Long axonal features representing both healthy and regenerating axons (green). Third row: Short axonal features representing degenerated axonal fragments and debris (red). Individual columns, moving left to right, correspond to different time points after neurorrhaphy. Scale bar is 50 μm.

As tissue clearing allows for the acquisition of the complete volumetric profile of axons throughout the nerve, a straightforward image analysis routine could be applied to analyze microscale changes and quantify Wallerian degeneration and axonal regeneration. Given the depth penetration provided by tissue clearing, peripheral nerve regrowth could be analyzed in three-dimensions, allowing the first visualization of axons in this environment outside of the central nervous system. High resolution volumes measuring 200×200×24 μm^3^ (32 sets/field) were taken from regions of stained, tissue cleared nerves, z-stacked, sorted into individual components, then scored using a Matlab script based on image segmentation. Randomized locations 1∼2 mm distal from the suture site in nerves harvested at timepoints pre-, 3, 6, 9 and 12 days post-operatively. Binary two-dimensional images were created and thresholded to eliminate background noise. The individual features (axonal segments) contained in the images were then analyzed and labeled as either long (representing healthy axons) depicted in green (Fig. 6, second row) or short (representing degenerating axons) depicted in red (Fig. 6, third row). It is worth noting that in this analysis, long features can represent both healthy and regenerating axons, which are both recognized as continuous and intact, although regenerating axons are consistently thinner than healthy or degenerating axons (Fig S2), a phenomenon known in previously published literature [36].

**Figure 7:**
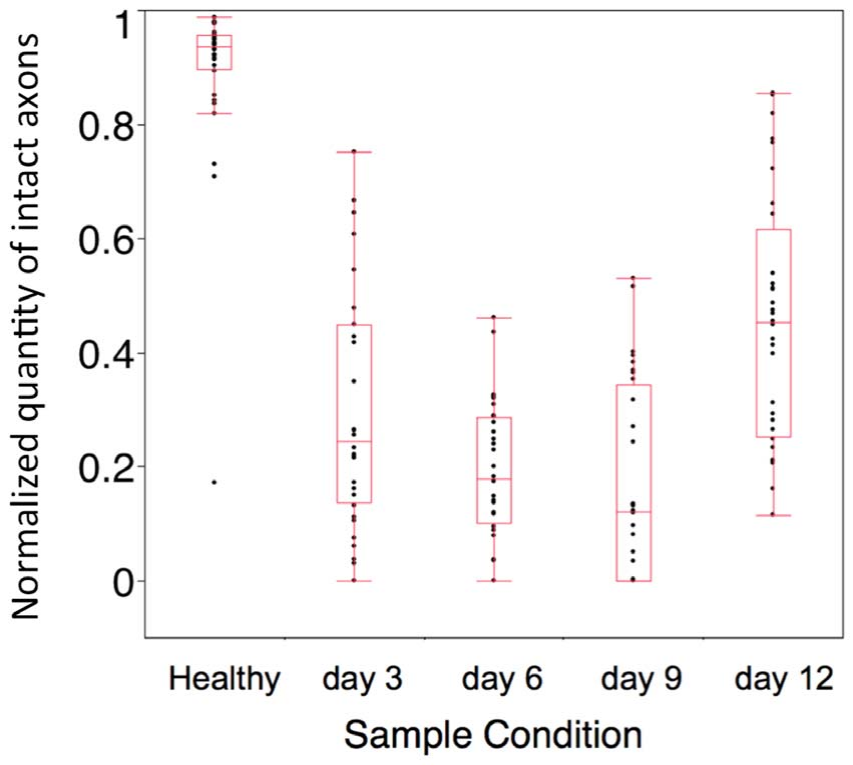
Normalized length scores for transected nerves at time points following surgical repair. The normalized value describing the amount of long axons was acquired from 32 datasets (200×200×24 μm^3^) at each time point taken 5 mm distal to the repair site, and indicated as black dots.

**Figure 8:**
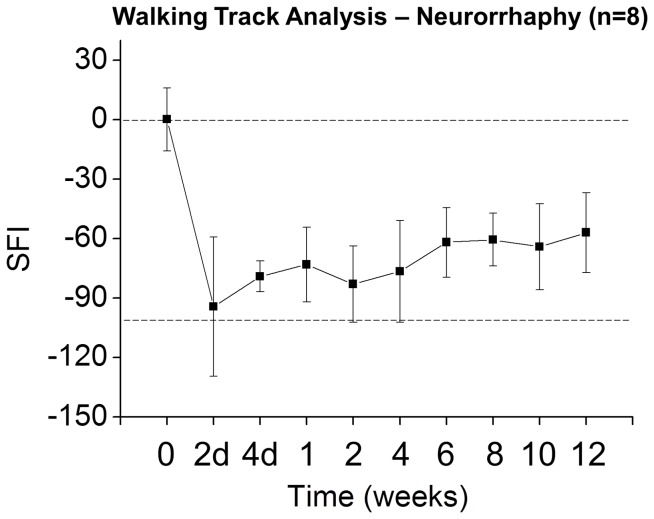
Example of walking track analysis obtained in wild type male Sprague Dawley rats after neurorrhaphy. Consistent with published literature [35], the animal reaches approximately 60% of baseline function by 12 weeks with a peripheral nerve growth rate of approximately 3 mm per day [36]. The sciatic functional index (SFI), a standard measure of functional recovery, is plotted along the y-axis, with a score of 0 representing a normal healthy nerve at baseline (top dotted line) and a score of −100 representing complete transection (bottom dotted line) [36].

To obtain an approximate ratio of healthy or regenerating axons to degenerating axons at each time point, the total area of long intact axons (green) in each image was counted, and normalized to the total area of all individual features in each image. All analyses were performed on image data acquired at a distance approximately 2 mm distal to the repair site. This was then plotted along a time course (Fig. 7) with a normalized length score of 1 representing healthy axons. At baseline, the normalized length score is 1 as the majority of axons have not yet been injured. This score significantly falls 72 hours after neurorrhaphy as Wallerian degeneration ensues, continues to fall throughout the first week, and reaches a minimum at 9 days before improving to 0.4–0.5 by day 12. This analysis is not only consistent with our qualitative observations noted in Figure 5, but suggested by observed functional recovery of a thy-1 GFP rat sciatic nerve after neurorrhaphy in both our laboratory (Fig. 8) and in the published literature [35], [36].

### Myelin Degeneration and Axonal Recovery

**Figure 9:**
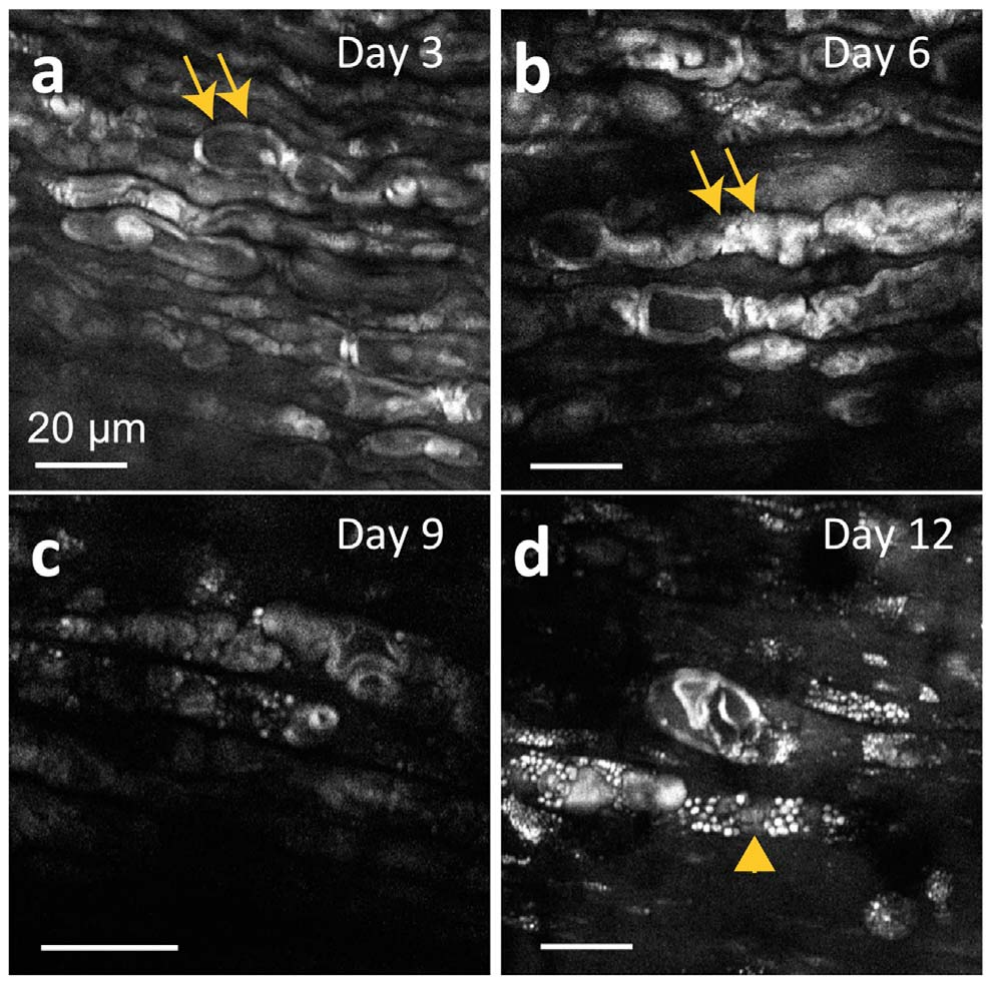
Time course CARS microscopy of distal sciatic nerves following neurorrhaphy. (a) At 3 days postoperatively, the myelin sheath anatomy suggests underlying structural chromatolysis and swelling of axons, continuing through day 6 (b). (c) At 9 days postoperatively, very little myelin signal is visualized as axons reach the nadir of degradation; macrophage-like cells with dark central nuclei begin to appear. (d) By 12 days postoperatively, lipid phagocytosis and possible metamorphosis of Schwann cells is observed. Double arrows denote myelin sheaths at early time points; the arrowhead denotes a macrophage-like cell at later time points.

Myelin, the lipid rich sheath that allows for rapid and reliable nerve signal conduction across large physical distances, is known to undergo changes during nerve healing, and has been followed in recent work with crush injuries [19], [20]. To investigate the changes in myelination that accompany degeneration and regrowth, we observed regrowth following neurorrhaphy, focusing on visualizing myelin in the healing nerves using CARS imaging. As the tissue clearing protocol actively removes lipids from tissue, CARS microscopy was performed on the same fresh *ex vivo* nerves used for the time course study (Figs. 5–7) prior to clearing. CARS microscopy is a label-free, chemically-selective imaging method that enables rapid assessment of tissue without the need for perturbative fixation or staining methods. CARS microscopy is exquisitely sensitive to lipids, enabling high-resolution imaging of myelin. While CARS microscopy is carried out here *ex vivo*, it is possible to carry out CARS imaging after minimum surgery *in vivo* as demonstrated in previous studies [16], [38]. Images of the myelin sheaths taken at the same time points as the confocal images in Figure 5 reveal the swelling and chromatolysis of myelin sheaths that take place after neurotmesis (Figs. 9a, 9b). Cells morphologically identified as macrophages [20], [37], [38] with dark central nuclei were observed to overrun the axons by nine days following transection (Fig. 9c), with lipid phagocytosis occurring at 12 days (Fig. 9d).

These results clearly demonstrate that axonal degeneration and regeneration preceded the completion of myelin degradation. Regeneration through the repair site was observed at 6 days postoperatively (Fig. 6), but regeneration of myelin was not observed even by 12 days postoperatively (Fig. 9). This finding is consistent with the improvements in electrophysiological and functional recovery that continued to occur long after axonal regeneration had taken place (Figure 8) [20], [32], [33], [39], [40].

## Discussion

While the prevalence of civilians suffering from peripheral nerve injury (PNI) has remained consistent over the past several decades, the exponential rise in extremity combat injuries over the last decade continues to pose a steady and concerning challenge for both the surgical and research communities. In the era of modern guerrilla warfare, dominated by deployment of improvised explosive devices (IEDs), the importance of discovering advances in peripheral nerve repair is more important than ever. Surgical success at saving both life and limb has increased incrementally with each major war during the 20^th^ and now 21^st^ century. Unfortunately, the surgical ability to repair injured peripheral nerves has not advanced in a similar fashion and slow painful recovery, enduring disability, and diminished quality of life remain the standard outcome. However, the potential to improve outcomes after PNI, unlike injuries of the central nervous system (CNS), remains vast, and advances in PNI research have the potential to yield immediate clinically applicable results due to the inherent regenerative capacity of peripheral nerves. Our current inability to visualize how axons regenerate throughout peripheral nerves is a fundamental gap in our research toolkit, essentially blinding researchers to the microscale details of the recovery process.

The tissue clearing, staining, and imaging approach described here offers an improved method that enables precise spatial imaging of axons with molecular contrast throughout nerves and within suture sites. The combination of whole mount immunofluorescence staining, tissue clearing, and confocal microscopy allowed for the visualization of largely unperturbed nerve bundles after neurorrhaphy. Immunofluorescence staining, followed by tissue clearing, enabled the tracing of individual axons hundreds of micrometers deep in tissue (Video S4). Axons in the center of the nerve were observed to have relatively weaker fluorescence than those found at the periphery. This difference in fluorescence intensity is thought to be a consequence the limited penetration depth of both primary and secondary antibodies during the relatively short incubation periods (3 days and one day, respectively). Longer incubation durations should allow for greater penetration of antibodies into tissue for improved fluorescence staining throughout the nerve. In contrast, tissue cleared, thy-1 GFP rat nerves were found to have consistent fluorescence intensity at all depths (Video S5a).

The significantly improved depth penetration following tissue clearing and the high resolution afforded by confocal microscopy importantly revealed both axonal connectivity and the ability to observe the trajectories of individual axons during regeneration, even across the repair site (Figure S3, Video S5b). Additionally, the ability to visualize regeneration concurrent with degenerating debris allowed for quantification of axonal regeneration using a straightforward analysis routine. This was demonstrated to be possible in both wild type, antibody-labeled nerves, as well as those excised from transgenic GFP rats, indicating that these protocols are likely applicable to many animal models.

The visualization of myelin in nerves is important in the monitoring and study of regenerating peripheral nerves following injury, which is significantly slowed as Schwann cells become senescent [33]. It is known that myelin degradation follows axonal degradation, with regeneration lagging behind axonal growth [20], [34], [40] This was readily observed using CARS microscopy in our neurorrhaphy model, with axonal regeneration occurring 12 days post-operatively, while myelin degeneration and macrophage invasion were still in progress (Figure S4). Time course studies have been planned to further follow the interplay between axonal regrowth, myelination, and behavioral characteristics. It is worth noting that this qualitative observation of Wallerian degeneration and regeneration is consistent with prior studies evaluating the effectiveness of the thy-1 GFP rat as a peripheral nerve injury model [12], [13], again suggesting that quantitative analysis can serve as an adjunct to current outcomes measurements. The results of this study suggest Wallerian degeneration occurring within 3 days and still present at 9 days post-injury, consistent with prior work that concludes Wallerian degeneration to complete by 14 days after axotomy or Sunderland Class V injury. This differs from observation in thy-1 YFP mice models, which have more rapid degenerative and regenerative rates due shorter axonal lengths [41], [42].

Despite the complexity of axonal degradation and regrowth, the rich structural information contained in the comprehensive 3D image volumes acquired from optically cleared tissue by confocal microscopy could readily be quantified using straightforward algorithms. This is a distinct advantage of volumetric imaging approaches: the data within large-area, stitched volumes can readily be mined with widely accessible and cost-effective software like Matlab and ImageJ. Although commercially available software tools exist that can trace individual nerve fibers and axonal counts [41], [42], including Amira (Visage Imaging, Version 5.3), Neurolucida (MBF Bioscience), and FilamentTracer (Bitplane scientific software), they are often expensive and complex. Moreover, these toolkits do not currently have the built-in ability to evaluate the degree of degeneration or regeneration in thousands of individual axons within in a single nerve fiber.

The ability to directly visualize nerve bundles branching into fascicles by virtual sectioning and scanning from proximal to distal reveals axonal connectivity otherwise lost with histology or indirect measurements of recovery. Histological processing requires the destructive manipulation of harvested nerve segments, leading to difficulty in reassembling and assessing longitudinal and large-scale axonal organization and connectivity. Additionally, the analysis of obtaining axonal counts and myelin thickness can be labor-intensive. On the contrary, the analysis of large-volume, high-resolution data stacks can be automatically processed over minutes using image analysis routines. As this approach offers significantly greater structural and connectivity information than standard histological preparation, it is thought that tissue clearing and imaging can not only increase the speed at which peripheral nerve research is performed, but can supplement outcomes measurements. Future quantitative outcomes experiments could utilize methods such as light sheet microscopy [43] to obtain high-resolution cross-sectional data comparable to the gold standard measurements provided by histology.

Additionally, this mapping of axonal connectivity will be useful for tracing individual axons as they potentially re-innervate distal endoneurial tubes, perhaps providing insight into neurotropism observed for both sensory and motor neurons [43]–[45]. Moreover, if true functional recovery following PNI requires re-innervation of the neuromuscular junction, future studies utilizing tissue clearing can focus on evaluating and quantifying axonal connectivity at the neuromuscular junction level in the muscle. Ultimately, if single axons can be traced over long nerve lengths, an axonal regeneration map could be created from dorsal root ganglia to end-target muscles, bridging the gap between indirect outcomes metrics and detailed microscale nerve imaging.

## Conclusions

In this report, we demonstrated an improved approach for comprehensive evaluation of whole sciatic nerves with subcellular resolution after neurorrhaphy. The combination of *ex vivo* tissue clearing following whole mount immunofluorescence staining and confocal microscopy enabled the direct visualization and analysis of the complex three-dimensional axonal structure in both healthy and surgically repaired nerves in the acute healing phase after PNI. This *ex vivo* analysis approach provides a new window for both researchers and clinicians into the microscale details of axonal regeneration after insult, where the paths and connectivity of individual axons can be identified and mapped. These methods have the ability to enhance PNI research and development, bridging the gap between outcomes metrics and the microscale details of nerve regeneration.

## Supporting Information

Figure S1 **The dimensions of individual axons within healthy rat sciatic nerves obtained from different imaging and sample preparation methods.** (a) Myelin sheaths visualized by CARS microscopy. (c) Axons genetically labeled by thy-1 GFP and visualized by confocal microscopy. (e) Axons labeled by anti-neurofilament antibodies visualized by confocal microscopy. (g) Axons tissue cleared following anti-neurofilament antibody labeling and visualized by confocal microscopy. (b, d, f, h) Intensity profiles along the yellow lines in (a, c, e, g), respectively. Sizes of axons were estimated based on the intensity profile. In Fig S1a and S1b, the size of the axon was measured as the distance between the ends of myelin sheaths surrounding axons.
(TIF)

Figure S2 **Degenerating and regenerating axons in stained, tissue cleared sciatic nerves.** (a, c) High resolution *ex vivo* images of the individual axons from wild type rat sciatic nerves that have undergone whole mount immunofluorescence staining and tissue clearing at 3 and 12 days post-neurorrhaphy, obtained using confocal microscopy. (b, d) Intensity profiles of the individual axons selected at the yellow line in (a,c), respectively. While the degenerating axons at 3 days post-neurorrhaphy showed discontinuity, the regenerating axons at 12 days post-neurorrhaphy were found to be continuous throughout the whole field of imaging.
(TIF)

Figure S3 **Individual axons traced across the repair site in tissue cleared thy-1 GFP rat sciatic nerves.** Axons are traced proximal (left) to distal (right) at 21 days post-neurorrhaphy. (a) Green lines depict individual axons successfully crossing the repair site; violet lines depict axons that fail to do so. (b) Reconstruction of a peripheral nerve with previously traced axons in 3D space. The dotted yellow line references the transverse cross sectional boundary of the nerve. Scale bar is 200 μm. Please see Appendix S1 for the axonal tracing method.
(TIF)

Figure S4 **Simultaneous evaluation of axons and myelin sheaths.** Images were taken in the sciatic nerve of a transgenic thy-1 GFP rat after neurorrhaphy approximately 1 mm distal to the repair site. (a,c) Confocal image of axons 2 and 4 days postoperatively, respectively. (b,d) CARS images of myelin sheaths 2 and 4 days postoperatively, respectively. Please see Appendix S1 for detailed methods describing the acquisition of this figure.
(TIF)

Appendix S1 **Axonal tracking and simultaneous CARS/confocal imaging supporting information.**
(DOCX)

Video S1 **a, b. Tissue clearing of the sciatic nerve of a wild-type rat after immunofluorescence staining.** (a) Longitudinal cross sectional *ex vivo* images as deep as 845 μm could be acquired after tissue clearing. (b) A three dimensional *ex vivo* image of the healthy sciatic nerve was reconstructed using acquired series of longitudinal cross sectional images. In this three-dimensional image, the nerve surface (epineurium), fascicles, and whole individual nerve fibers deep in the nerve could be seen.
(MP4)

Video S2 **Transverse cross section of a sciatic nerve without physical sectioning.** The continuous cross sectional *ex vivo* image of a healthy rat sciatic nerve is visualized after immunofluorescence staining and tissue clearing. The video starts from the proximal site of the sciatic nerve and progress to the distal site. In the video, the division of 3 fascicles into 6 distinct fascicles can be observed. This video covers approximately 3.6 mm of the sciatic nerve. In the video, not only fascicles and nerve fibers but also blood vessels can be observed.
(MP4)

Video S3 **a–d. Time course study of nerve regeneration.** (a–d) High resolution, large area, three dimensional *ex vivo* images of rat sciatic nerves following neurorrhaphy are shown at 3, 6, 9 and 12 days postoperatively. Sutures are identified by their dark contrast in the images. Proximal and distal sides are located to the left and right of the sutures, respectively. Red in the image represents nearly saturated signal.
(MP4)

Video S4 **a–c. Tracing individual nerve fibers located deep within rat sciatic nerves.** (a) Longitudinal cross sectional *ex vivo* images. The size of image is 211×211×100 μm^3^. Several individual axons were selected and displayed as green to demonstrate the capability to trace individual axons located deep in the tissue after tissue clearing. (b) A three dimensional *ex vivo* high resolution image of the healthy sciatic nerve was reconstructed using acquired series of longitudinal cross sectional images in (a). By changing the transparency of the image, the selected several individual axons with green color can be seen in three dimensional structural of the nerve bundle. (c) Rotation of the three dimensional image of the nerve fibers.
(MP4)

Video S5 **a,b. Tracing individual nerve fibers across the repair site.** (a) *Ex vivo* longitudinal cross sectional image of thy-1 gfp rat sciatic nerve 22 days after neurorrhaphy. Proximal and distal sides are located to the left and right sides of the video, respectively. (b) Visualizing the individual axons crossing the repair site in three-dimensional space.
(MP4)

Checklist S1 **ARRIVE guidelines checklist.**
(DOC)
