# Supplementary figures and images for: Comprehensive Evaluation of Peripheral Nerve Regeneration in the Acute Healing Phase Using Tissue Clearing and Optical Microscopy in a Rodent Model
Source: PLoS One. 2014 Apr 8;9(4):e94054. doi: 10.1371/journal.pone.0094054 (PMC3979924; doi:10.1371/journal.pone.0094054)

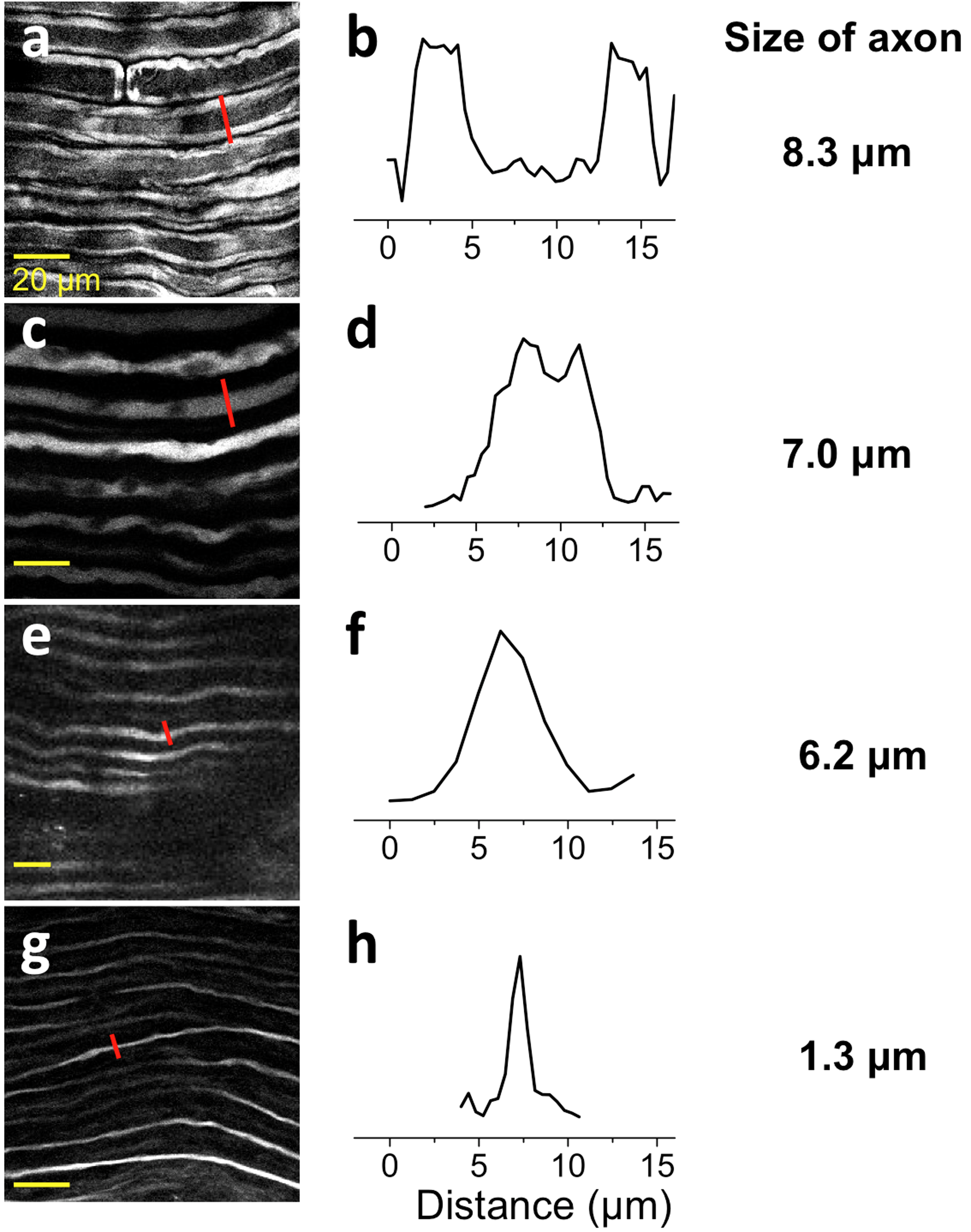

Supplement: Figure S1 — The dimensions of individual axons within healthy rat sciatic nerves obtained from different imaging and sample preparation methods. (a) Myelin sheaths visualized by CARS microscopy. (c) Axons genetically labeled by thy-1 GFP and visualized by confocal microscopy. (e) Axons labeled by anti-neurofilament antibodies visualized by confocal microscopy. (g) Axons tissue cleared following anti-neurofilament antibody labeling and visualized by confocal microscopy. (b, d, f, h) Intensity profiles along the yellow lines in (a, c, e, g), respectively. Sizes of axons were estimated based on the intensity profile. In Fig S1a and S1b, the size of the axon was measured as the distance between the ends of myelin sheaths surrounding axons. (TIF) [file pone.0094054.s001.tif]

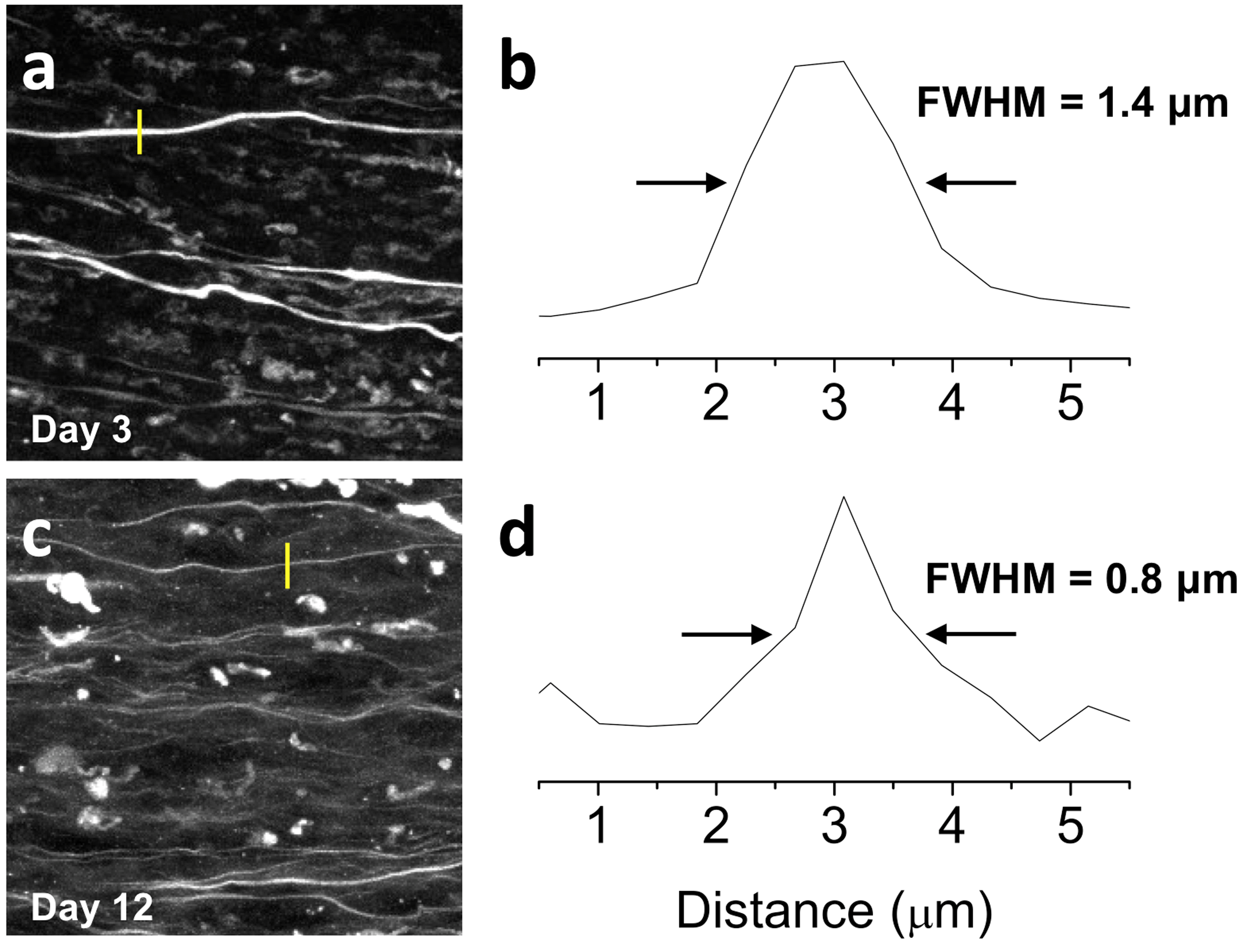

Supplement: Figure S2 — Degenerating and regenerating axons in stained, tissue cleared sciatic nerves. (a, c) High resolution ex vivo images of the individual axons from wild type rat sciatic nerves that have undergone whole mount immunofluorescence staining and tissue clearing at 3 and 12 days post-neurorrhaphy, obtained using confocal microscopy. (b, d) Intensity profiles of the individual axons selected at the yellow line in (a,c), respectively. While the degenerating axons at 3 days post-neurorrhaphy showed discontinuity, the regenerating axons at 12 days post-neurorrhaphy were found to be continuous throughout the whole field of imaging. (TIF) [file pone.0094054.s002.tif]

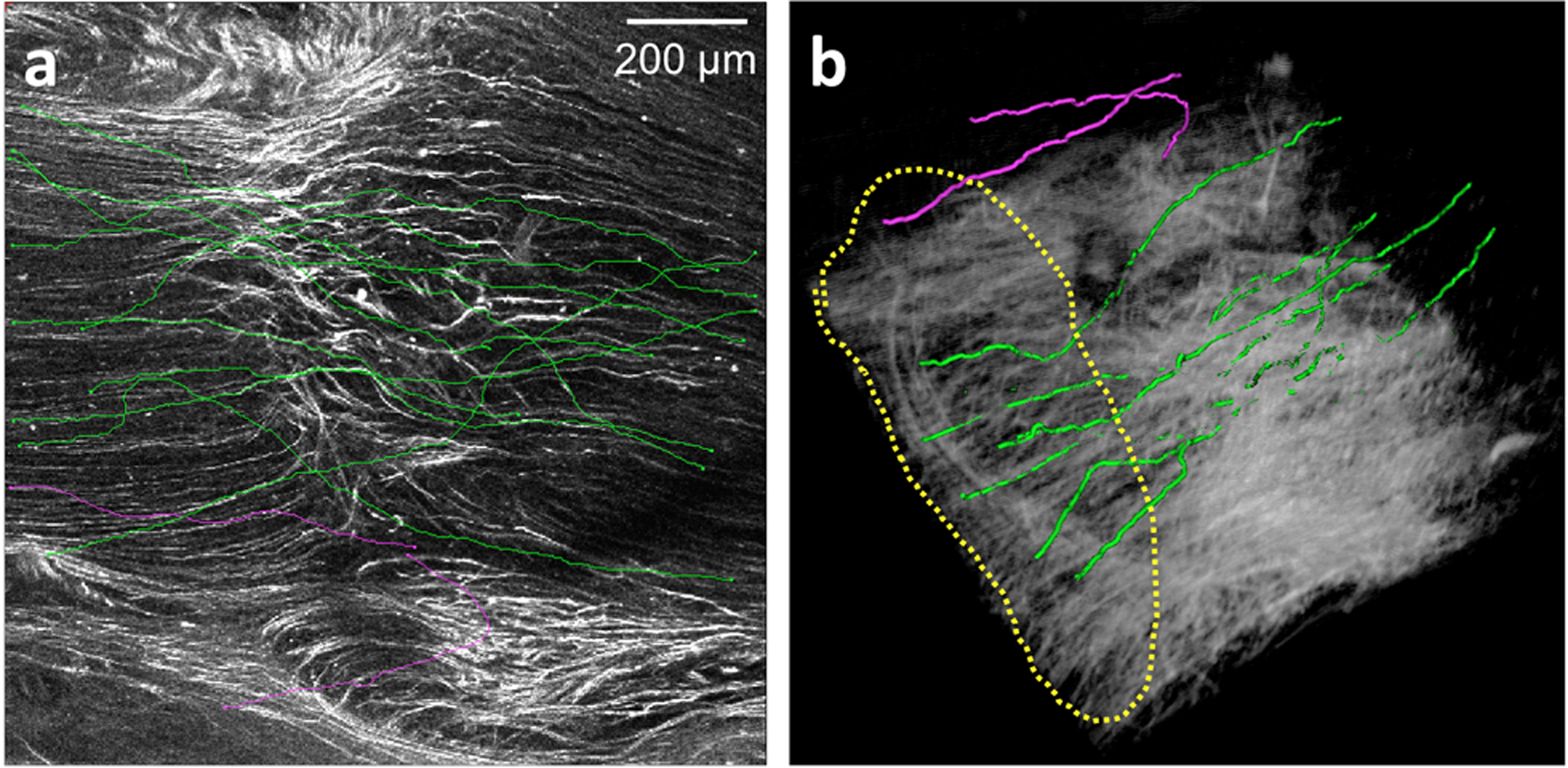

Supplement: Figure S3 — Individual axons traced across the repair site in tissue cleared thy-1 GFP rat sciatic nerves. Axons are traced proximal (left) to distal (right) at 21 days post-neurorrhaphy. (a) Green lines depict individual axons successfully crossing the repair site; violet lines depict axons that fail to do so. (b) Reconstruction of a peripheral nerve with previously traced axons in 3D space. The dotted yellow line references the transverse cross sectional boundary of the nerve. Scale bar is 200 μm. Please see Appendix S1 for the axonal tracing method. (TIF) [file pone.0094054.s003.tif]

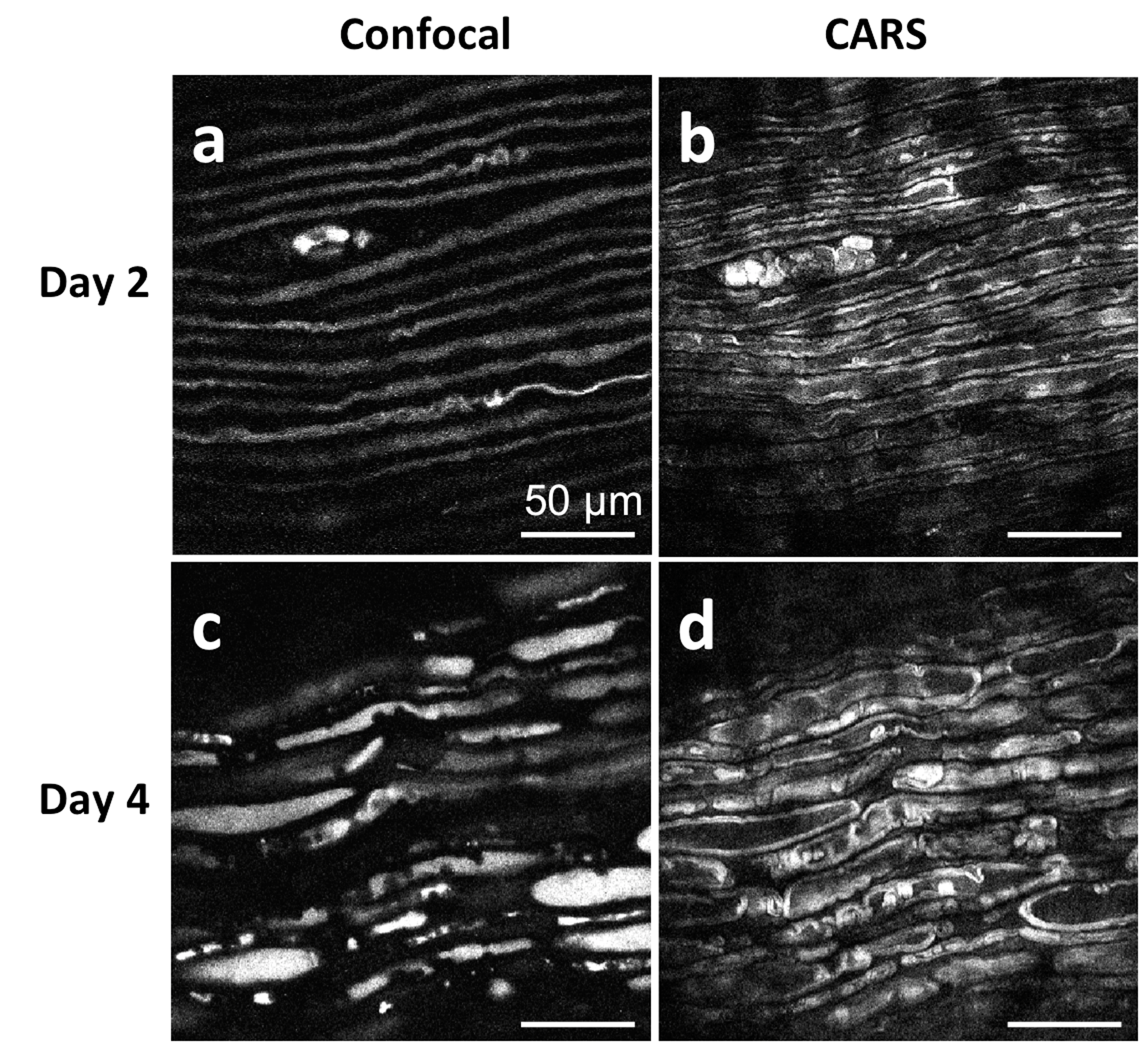

Supplement: Figure S4 — Simultaneous evaluation of axons and myelin sheaths. Images were taken in the sciatic nerve of a transgenic thy-1 GFP rat after neurorrhaphy approximately 1 mm distal to the repair site. (a,c) Confocal image of axons 2 and 4 days postoperatively, respectively. (b,d) CARS images of myelin sheaths 2 and 4 days postoperatively, respectively. Please see Appendix S1 for detailed methods describing the acquisition of this figure. (TIF) [file pone.0094054.s004.tif]
