# Supplementary material for: Comprehensive Evaluation of Peripheral Nerve Regeneration in the Acute Healing Phase Using Tissue Clearing and Optical Microscopy in a Rodent Model
Source: PLoS One. 2014 Apr 8;9(4):e94054. doi: 10.1371/journal.pone.0094054 (PMC3979924; doi:10.1371/journal.pone.0094054)
Supplement: Appendix S1 — Axonal tracking and simultaneous CARS/confocal imaging supporting information. (DOCX) [file pone.0094054.s005.docx]

**Appendix: Supporting Information**

**Axonal tracing across the repair site**

Following injury, neurotrophic factors and signals stimulate regenerating axons to grow towards formerly occupied endoneurial tubes in the distal nerve [[1](#_ENREF_1)]; however, the actual direction in which axons travel to reach distal targets can be difficult to evaluate. From a qualitative perspective, time post-injury aside, the observation of well-aligned, intact axons distal to the injury site is a strong indicator that re-innervation was successful; however the correct topographical alignment of axons back to specific distal targets is what allows for functional motor and sensory recovery. Axons that do not regenerate through the repair site or take an eccentric pathway create painful and non-functional outgrowths called neuromas; axons that successfully regenerate further distally may be subject to spatial and ultimately functional misdirection. Understanding the detailed, three-dimensional re-growth pattern and axonal directionality of individual axons following various types of repair raises interesting questions about spatial organization of axons and may assist in optimizing future surgical protocols.

To demonstrate the ability to track individual axons regenerating through the repair site in tissue-cleared nerves, a sciatic nerve was harvested from a transgenic thy-1 GFP rat 21 days post neurorrhaphy. A three-dimensional optical image of the whole nerve at the repair site was acquired after tissue clearing (Supplementary Video 1). Individual axons from the acquired 3D image were traced across the repair site (Fig. S3). The ‘Simple Neurite Tracer’ plugin, an open source image plugin included in the Fiji software collection, was used to trace unique axons [[2](#_ENREF_2)]. Traced axons were labeled in various colors for contrast, before being overlaid on the tissue cleared repair site image. Axons that successfully crossed the repair site were labeled in green, while those that did not cross the repair site are labeled in violet (Fig. S3a). The individually traced axons could then be visualized within the nerve bundle in three-dimensional space (Fig. S3b and Supplementary Videos 4a,b,c and 5).

**Simultaneous imaging of both axons and myelin using a GFP rat model**

The transgenic thy-1 GFP rat model has become very useful for studying PNI with optical microscopy [[3](#_ENREF_3),[4](#_ENREF_4)]. Combining CARS microscopy with confocal imaging of the transgenic rats provides simultaneous comparison of axonal and myelin alterations within the same nerve. In Figure S4, axons (Figs. S4a, S4c) were imaged with confocal microscopy, and myelin sheaths (Figs. S4b, S4d) were imaged with CARS microscopy. These images were obtained in freshly harvested nerves two (Figs. S4a, S4b) and four days (Figs. S4c, S4d) postoperatively, and demonstrate axonal degeneration preceding myelin sheath metamorphosis. This known lag in myelin regeneration is thought to occur due to the loss of the axonal cytoskeleton that serves as a myelin sheath scaffold [[5](#_ENREF_5)] (Fig. S4d). Macrophage invasion and lipid phagocytosis are not observed at these early time points.

**References**

1. Misgeld T, Kerschensteiner M (2006) In vivo imaging of the diseased nervous system. Nature Reviews Neuroscience 7: 449-463.

2. Longair MH, Baker DA, Armstrong JD (2011) Simple Neurite Tracer: open source software for reconstruction, visualization and analysis of neuronal processes. Bioinformatics 27: 2453-2454.

3. Magill CK, Moore AM, Borschel GH, Mackinnon SE (2010) A New Model for Facial Nerve Research. Archives of Facial Plastic Surgery 12: 315-320.

4. Moore AM, Borschel GH, Santosa KA, Flagg ER, Tong AY, et al. (2012) A transgenic rat expressing green fluorescent protein (GFP) in peripheral nerves provides a new hindlimb model for the study of nerve injury and regeneration. Journal of Neuroscience Methods 204: 19-27.

5. Bélanger E, Henry F, Vallée R, Randolph M, Kochevar I, et al. (2011) In vivo evaluation of demyelination and remyelination in a nerve crush injury model. Biomedical optics express 2: 2698.
